# Integrin β‐3 is required for the attachment, retention and therapeutic benefits of human cardiospheres in myocardial infarction

**DOI:** 10.1111/jcmm.13325

**Published:** 2017-10-11

**Authors:** Suyun Liu, Zhian Jiang, Li Qiao, Bingyan Guo, Wenliang Xiao, Xiaoguang Zhang, Liang Chang, Yongjun Li

**Affiliations:** ^1^ The Second Hospital of Hebei Medical University Shijiazhuang China; ^2^ The Third Hospital of Hebei Medical University Shijiazhuang China

**Keywords:** cardiospheres, cardiac stem cells, integrin, myocardial infarction

## Abstract

Cardiovascular diseases remain the leading causes of death worldwide. Stem cell therapy offers a promising option to regenerate injured myocardium. Among the various types of stem cells, cardiosphere cells represent a mixture of intrinsic heart stem cells and supporting cells. The safety and efficacy of cardiosphere cells have been demonstrated in recent clinical trials. Cell–matrix interaction plays an important role in mediating the engraftment of injected stem cells. Here, we studied the role of integrin β‐3 in cardiosphere‐mediated cell therapy in a mouse model of myocardial infarction. Our results indicated that inhibiting integrin β‐3 reduced attachment, retention and therapeutic benefits of human cardiospheres in mice with acute myocardial infarction. This suggests integrin β‐3 plays an important role in cardiosphere‐mediated heart regeneration.

## Introduction

Cardiospheres (CSs) are one type of multicellular spheroids representing three‐dimensional culture of cardiac stem cells and other supporting cells 1, 2. Cardiosphere‐derived single cells (CDCs) are currently under phase II clinical investigation for patients with mild‐to‐moderate myocardial infarction 3, 4. Previous reports have demonstrated the importance of cell–cell contact in the therapeutic benefits of cardiosphere cells 5, 6, 7. When compared to their monolayer‐cultured counterparts CDCs, cardiospheres showed enhanced engraftment and therapeutic outcome for the treatment of ischaemic cardiomyopathy in mouse and pig model of acute myocardial infarction 8, 9, 10. In addition, previous studies revealed that genes related to adhesion molecules (*e.g*. integrin β‐3) and stemness (Nanog, Sox2) are up‐regulated in CSs as compared to CDCs 8, 10. Integrin β‐3 (ITB3) can interact with fibronectin (FN), which is abundant in the myocardium matrix 11. We hypothesize that ITB3 is essential to the adhesion of CSs in the myocardium therefore can govern the engraftment and therapeutic benefits of CSs. To test this hypothesis, we used a neutralizing ITB3 antibody to pre‐treat CSs and then evaluated their attachment potency *in vitro*, and engraftment and therapeutic benefits in the heart in a mouse model of AMI.

## Materials & methods

### Derivation and culture of CSs from human hearts

Human CSs were derived as previously described 12. In brief, heart tissues were minced into small pieces about 1–2 mm^3^, then washed with PBS and digested with collagenase solution for 15 min. (Sigma‐Aldrich, St. Louis, MO, USA), the tissue fragments were cultured as “cardiac explants” on plate coated with 0.25 mg/ml fibronectin (BD Biosciences, San Jose, CA, USA) in Iscove's modified Dulbecco's medium (IMDM; Invitrogen, Carlsbad, CA, USA). The IMDM media was supplemented with 20% foetal bovine serum (FBS; Corning, Corning, NY, USA), 0.5% Gentamicin (Gibco, Life Technologies, Durham, CA, USA), 0.1 mM 2‐mercaptoethanol (Invitrogen) and 1% L‐glutamine (Invitrogen). In about 1–2 weeks, a layer of stromal‐like flat cells, and phase‐bright round cells, emerged from the cardiac explant with phase‐bright cells over them. These cardiac explant‐derived cells were harvested using TryPEL Select (Gibco) and then seeded at a density of 2 × 10^4^ cells/ml in poly‐d‐lysine‐coated flaks for cardiosphere formation. In about 3–5 days, explant‐derived cells spontaneously aggregated into cardiospheres. The morphology of cardiospheres was checked with a Nikon phase contrast microscope.

### Characterization of human CSs

Cardiospheres were incubated with fluorophore‐conjugated primary antibodies against CD105, CD90, ckit (positive markers for CSs) and CD45, CD31, CD34 (negative markers for CSs). Primary antibodies were obtained from Abcam (Cambridge, United Kingdom) or R & D Systems (Minneapolis, MN, USA) and BD Biosciences and used with dilutions recommended by the vendor. FITC‐ or Texas‐Red‐conjugated secondary antibodies were purchased from the same vendors. The samples were imaged by a fluorescent microscope.

### ITB3 inhibition on CSs and toxicity assay

Human CSs were incubated with anti‐ITB3 antibodies (10 μg/ml) for 1 hr at 37°C. After that, cell viability in the CSs was evaluated by EthD staining (Live/Dead assay kit, Invitrogen). Cell nuclei were counter‐stained with Hoechst (Invitrogen). The images were taken with an Olympus Confocal Microscope, and the percentage of EthD negative (live) cells were quantified. CS numbers and sizes were monitored over the time to evaluate the impact of ITB3 antibody treatment.

### *In vitro* attachment assay

Human CSs were treated with anti‐ITB3 or isotype control antibodies (10 μg/ml) for 1 hr at 37°C. After that, CSs were plated onto fibronectin‐coated surfaces for CS attachment. At 10, 20, 40 and 60 min. after plating, non‐attached CSs were removed and the percentage of attached CSs were quantified using a phase‐bright microscope.

### Mouse model of acute myocardial infarction (AMI)

The method to induce myocardial infarction in mice was based on previous studies 13. Briefly, male SCID mice were anaesthetized with 3% isoflurane combined with 2% oxygen inhalation. Under sterile conditions, the heart was exposed by a minimally invasive left thoracotomy, and acute myocardial infarction (MI) was produced by permanent ligation of the left anterior descending coronary artery. Immediately after AMI induction, the heart was randomized to receive one of the following three treatment arms: (*i*) AMI + CS + ITB3 ab: intramyocardial injection of 1 × 10^5^ cell‐formed CSs pre‐treated with ITB3 antibodies in 50 μl PBS into the heart immediately after MI; (*ii*) AMI + CS + control ab: 1 × 10^5^ cell‐formed CSs pre‐treated with control antibodies in 50 μl PBS into the heart immediately after MI; (*iii*) AMI + PBS: intramyocardial injection of 50 μl PBS into the heart immediately after MI. To enable visualization of injections in a cohort of animals, we pre‐labelled the CSs CM‐DiI (1 mg/ml [Invitrogen]). Cells were injected into four sites in the peri‐infarct area of the heart.

### CS retention assay by *ex vivo* fluorescent imaging

To enable fluorescent imaging and histological detection, CSs were pre‐labelled with red fluorophore DiI. Twenty‐four hours after injection, mice were killed to harvest the heart. *Ex vivo* fluorescent imaging was performed with an IVIS Xenogen *In Vivo* Imager (Caliper Lifesciences, Waltham, MA, USA).

### CS retention assay by quantitative PCR

Animals were killed, and their hearts excised to obtain an actual measurement of the number of cells engrafted. Real‐time PCR experiments using the human‐specific repetitive Alu sequences were conducted. The whole heart was weighed and homogenized. Genomic DNA was isolated using the DNAeasy minikit (Qiagen, Hilden, Germany). The TaqMan^®^ assay (Applied Biosystems, Foster City, CA, USA) was used to quantify the number of transplanted cells with the human Alu sequence as template (Alu forward, 5′‐CAT GGT GAA ACC CCG TCT CTA‐3′; Alu reverse, 5′‐GCC TCA GCC TCC CGA GTA G‐3′; TaqMan probe, 5′‐FAM‐ATT AGC CGG GCG TGG TGG CG‐TAMRA‐3′, Applied Biosystems). For absolute quantification of cell number, a standard curve was generated with known numbers of human cells.

### Cardiac function assessment

The transthoracic echocardiography procedure was performed by an animal cardiologist blind to the experimental design using a Philips ultrasound system. All animals underwent inhaled 1.5% isoflurane–oxygen mixture anaesthesia in supine position at the 4 hrs and 3 weeks. Hearts were imaged 2D in long‐axis views at the level of the greatest LV diameter. EF was determined by measurement from views taken from the infarcted area.

### Measurement of scar size and viable myocardium

After the echocardiography study at 3 weeks, animals were killed and hearts were harvested and frozen in OCT compound. Specimens were sectioned at 10 μm thickness from the apex to the ligation level with 100‐μm intervals. Masson's trichrome staining was performed as described by the manufacturer's instructions (Sigma‐Aldrich). From the Masson's trichrome‐stained images, morphometric parameters including viable myocardium and scar size were measured in each section with NIH ImageJ software. The percentage of viable myocardium as a fraction of the scar area (infarcted size) was quantified as described 14. Three selected sections were quantified for each animal.

### Histology

Heart cryo‐sections were fixed with 4% paraformaldehyde, permeabilized and blocked with protein block solution (DAKO, Carpinteria, CA, USA) containing 0.1% saponin (Sigma‐Aldrich) and then incubated with the following antibodies overnight at 4°C: mouse anti‐alpha sarcomeric actin (1:100, a7811, Sigma‐Aldrich) and human nuclei antigen (HNA) (1:200, Millipore, Billerica, MA), followed by incubation with Texas‐Red or FITC‐conjugated secondary antibodies. The images were taken by an Olympus epi‐fluorescence microscopy system.

### Statistical analysis

All results are expressed as mean ± standard deviation (S.D.) Comparison between two groups was conducted by two‐tailed Student's *t*‐test. One‐way anova test was used for comparison among three or more groups with Bonferroni post hoc correction. Differences were considered statistically significant when *P*‐values <0.05.

## Results

### Generation and characterization of human CSs

**Figure 1:**
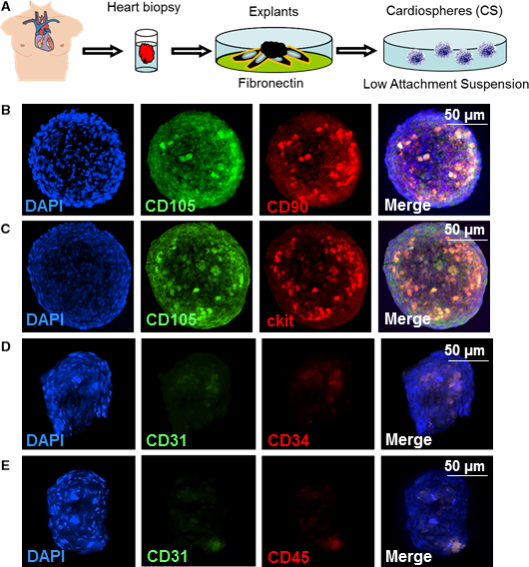
Antigenic phenotypes of the cells in cardiospheres. (**A**) Schematic showing the process of deriving cardiospheres from human myocardial tissue samples; (**B**) represent fluorescent micrographs showing the expressions of CD105 and CD90 in cardiospheres; (**C**) represent fluorescent micrographs showing the expressions of CD105 and ckit in cardiospheres; (**D**) represent fluorescent micrographs showing the expressions of CD31 and CD34 in cardiospheres; (**E**) represent fluorescent micrographs showing the expressions of CD31 and CD45 in cardiospheres. Scale bars = 50 μm.

The tissue processing is depicted in Figure 1A. In about 3–5 days, explant‐derived cells spontaneously aggregated into cardiospheres (CSs). Consistent with previous report, CSs were positive for CD105, CD90 and ckit (Fig. 1B and C), but are negative for CD31, 34 and 45 (Fig. 1D and E).

### Impact of ITB3 antibody treatment on CS viability and function

**Figure 2:**
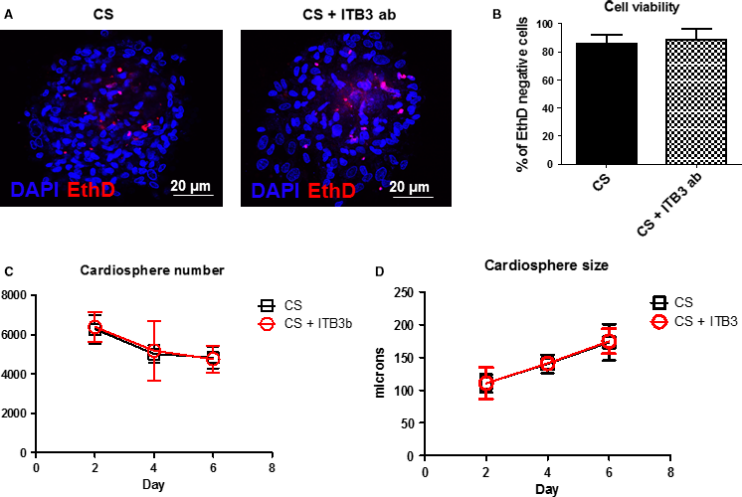
ITB3 inhibition is non‐toxic to cardiospheres. (**A**,** B**) Cell death staining (using EthD) reveals that ITB3 inhibition does not increase the numbers of dead cells in the cardiospheres. In addition, ITB3 inhibition does not affect cardiosphere numbers (**C**) and sizes over the time (**D**). Scale bars = 20 μm. *n* = 3 for each experiment.

Confocal microscopic imaging revealed a similar degree of cell death in ITB3 ab‐treated and control CSs (Fig 2A and B), indicating ITB3 antibody treatment did not affect the viability of cells in CSs. Moreover, CS numbers and sizes were indistinguishable between the two groups. These compound data sets confirmed the non‐toxicity of ITB3 antibody treatment on CSs.

### Inhibition of ITB3 reduces CS attachment potency and retention

**Figure 3:**
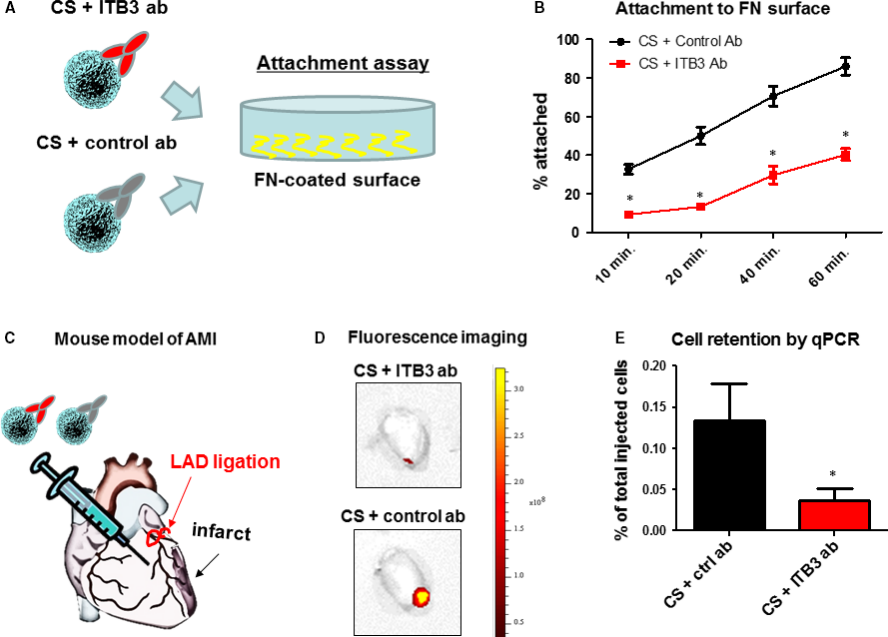
ITB3 inhibition reduces cardiosphere attachment *in vitro* and retention *in vivo*. (**A**) Schematic showing the attachment assay. (**B**) Treatment with ITB3 antibodies reduces cardiosphere attachment to fibronectin‐coated surface. (**C**) Schematic showing LAD ligation and intramyocardial injection of cardiospheres in mice. (**D**) *Ex vivo* fluorescent imaging showing DiI‐labelled cardiosphere retention in the heart. (**E**) Cell retention measured by sex‐mismatch PCR. *n* = 3 for each experiment. * indicated *P* < 0.05 when compared to the other group.

The attachment potency of ITB3‐inhibited and non‐inhibited CSs to FN‐coated surface was assessed (Fig. 3A). ITB3‐inhibited CSs had poor attachment to FN surface at all time‐points as compared to control CSs (Fig. 3B). We then tested the idea *in vivo* in a mouse model of AMI. Immunodeficiency SCID mice were used to avoid rejection of injected human cells. Equal numbers of ITB3‐inhibited and non‐inhibited CSs were directly injected into the mouse heart immediately after LAD ligation (Fig. 3C). *Ex vivo* heart fluorescent imaging 24 hrs later revealed larger signal (as an indicator of CS retention) in the heart for the control ab‐treated group (Fig. 3D). This was further confirmed by quantitative PCR evaluation of exact numbers of human cells in the mouse heart (Fig. 3E). These data sets indicate ITB3 inhibition diminishes CS retention in the heart, possibly though the blockage of ITB3‐FN interaction.

### Inhibition of ITB3 diminishes the therapeutic benefits of CSs in mice with AMI

**Figure 4:**
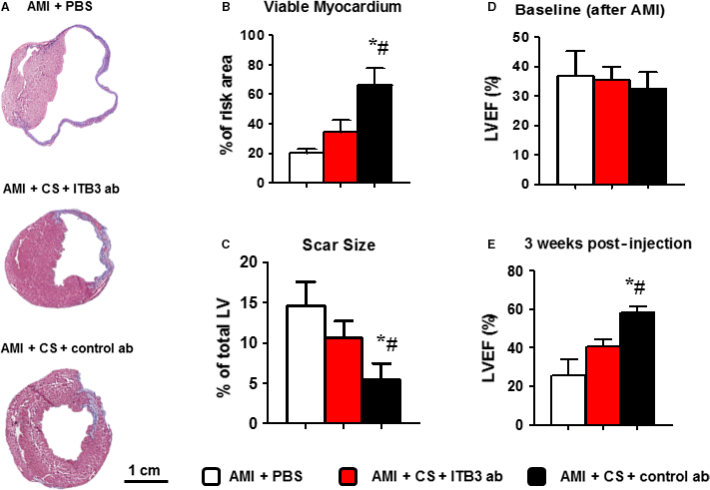
ITB3 inhibition blunts the functional benefits of cardiosphere treatment. (**A**) Masson's trichrome staining revealed scar (blue) versus healthy (pink) myocardium. (**B**,** C**) Cardiosphere treatment (black bars) effectively increases viable myocardium (**B**) but reduces scar size (**C**), as compared to vehicle control (white bars). Such benefits are abolished in the animals treated with ITB3‐inhibited cardiospheres (red bars). (**D**) LVEFs at baseline (4 hrs after MI) are indistinguishable among all groups. (**E**) 3 weeks after, the LVEFs for the control (white bar) and ITB3‐inhibited cardiosphere group (red bar) are still indistinguishable, while the cardiosphere‐treated group (black bar) exhibits larger LVEF. *n* = 5 animals per group. *indicated *P* < 0.05 when compared to the “AMI + PBS” group. # indicated *P* < 0.05 when compared to the “AMI + CS + ITB3 ab” group.

It was already known that CS treatment improves cardiac function in a mouse model of AMI 12. Here, we tested the hypothesis that the reduction in CS retention/attachment will ultimately harm the therapeutic outcome of CS treatment. Masson's trichrome staining enabled the visualization of scar (blue) and viable myocardium (red) (Fig. 4A). Consistent with prior reports 12, CS treatment increased viable myocardium (black bar *versus* white bar, Fig. 4B) but decreased scar size (black bar *versus* white bar, Fig. 4C). Interestingly, ITB3 inhibition blunted such benefits, as hearts injected with ITB3‐inhibited CSs failed to exhibit increased viable myocardium or decreased scar size (red bars, Fig. 4B and C). The ultimate bona fide indicator of cell therapies is the ability to preserve or improve cardiac function such as left ventricular ejection fraction (LVEF%) measured by echocardiography. LVEFs were indistinguishable among all groups at the baseline (4 hrs post‐AMI), indicating a similar degree of initial injury (Fig. 4D). Over the 3‐week time course, the LVEFs in control (PBS‐injected) animals deteriorated to ~20% (white bar, Fig. 4E). In contrast, control CS‐treated animals exhibited a boost of LVEFs (black bar, Fig. 4E). ITB3 inhibition resulted in disabled CS treatment which failed to improve cardiac functions (red bar, Fig. 4E).

### Inhibition of ITB3 impaired CS engraftment in the post‐MI heart

**Figure 5:**
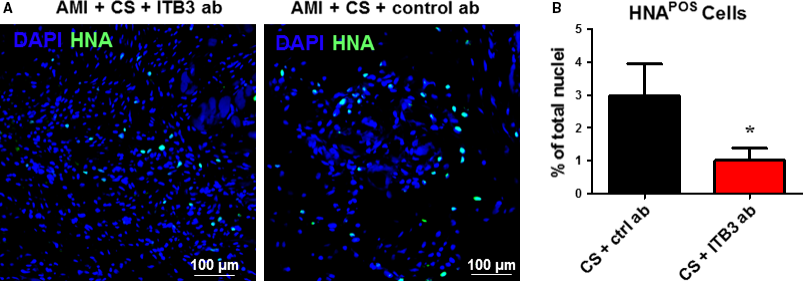
ITB3 inhibition impairs cardiosphere engraftment in the mouse heart. (**A**) Representative fluorescent micrographs showing HNA‐positive cells (green) in the mouse heart 3 weeks after injection. (**B**) Quantitation of the percentage of HNA‐positive cells. *n* = 3 for each experiment. *indicated *P* < 0.05 when compared to the other group. Scale bars = 100 μm.

To evaluate the impact of ITB3 inhibition on long‐term cell engraftment, we utilized human nuclei antigen (HNA) probes to detect human cells in the mouse heart. Histology revealed that at 3 weeks after injection, only negligible amounts of transplanted CS cells (labelled with HNA; green in Fig. 5A) were detected in the mouse myocardium. However, ITB3 inhibition reduced the engraftment of CS cells (Fig. 5B).

## Discussion

Integrins are transmembrane receptors that are the bridges for cell–cell and cell–extracellular matrix (ECM) interactions 10, 15. When triggered, integrins in turn trigger chemical pathways to the interior (signal transduction), such as the chemical composition and mechanical status of the ECM, which results in a response (activation of transcription) such as regulation of the cell cycle, cell shape and/or motility; or new receptors being added to the cell membrane. This allows rapid and flexible responses to events at the cell surface, for example to signal platelets to initiate an interaction with coagulation factors. Integrins are essential for stem cell functions as well 16, 17.

Cardiospheres (CSs) are natural three‐dimensional aggregates of heart‐derived cells. The therapeutic potential of CSs and CS‐derived cells (*i.e*. CDCs) has been demonstrated in numerous pre‐clinical studies 18 and recent clinical trials 3. Moreover, it has been reported that CSs are more potent for cardiac regeneration than CDCs in the same mouse model of AMI 8. This is due at least in part to the elevated expressions of stemness and ECM genes in CSs. Previous reports indicate that cardiospheres only express a low amount of ckit and ckit might not be important to the functions of cardiospheres.

Here, we tested the hypothesis that the therapeutic benefits of CSs require the involvement integrin β‐3 (ITB3) which is an important integrin interacts with fibronectin, the abundant form of ECM present in the heart and also the ECM used for the expansion of CS‐forming cells. In an *in vitro* attachment assay, it was obvious that blocking ITB3 significantly impaired the CSs’ ability to attach to FN‐coated surface (Figs 3 and B). This finding translated *in vivo* as ITB3‐blunted CSs fail to retain in the heart after delivery (Fig. 3C–E). The haemodynamic force generated by venous drainage made the heart not the ideal organ for cell retention/engraftment 19. In addition, the interaction between ECM and ITB3 may provide additional pro‐survival signals for cardiospheres *in vivo*.

The last decade has seen multiple strategies for enhancing cell retention in the heart, such as magnetically targeted cell delivery 20 and delivering cells in injectable hydrogels 21. Our results support the notion that enhanced cell retention can translate into augmented therapeutic benefits. In addition, short‐term cell retention is an excellent indicator for long‐term engraftment and functional benefit 19, 20. However, these physical strategies cannot detour the ultimate requirement for engraftment, which is the biological bonding between cells and the native ECM. We proved that the ITB3‐FN adhesion axis plays an essential role in CS engraftment (Fig. 5). The two functional benefit indicators of CS treatment are the attenuation of heart remodelling (assessed by Masson's trichrome staining and heart morphometry) and protection of cardiac function (assessed by echocardiography). Consistent with our previous studies, CS treatment led to a protection of both heart morphology and function (Fig. 4). However, the ITB3‐blunted CSs failed to generate the same therapeutic outcome. A previous report identified that the integrin beta 1 expressions on cardiomyocytes are essential to the therapeutic benefits of cardiosphere‐derived cells 5. Our study provides additional insights that multiple integrin types pay important roles in stem cell‐mediated heart regeneration. It has also been reported before that the presence of cardiac stem cells correlates with the expression of fibronectin during cardiac development and after MI 22. Genetic conditional ablation of fibronectin reduces stem cell response. Also, attenuated angiomyogenesis is evident. The mechanism involves fibronectin‐mediated protection and promotion of proliferation of stem cells *via* integrin beta 1. As integrin β‐3 also binds to fibronectin, out study provides further evidence that integrin–fibronectin interaction is essential to stem cell‐mediated repair.

Mounting lines of evidence indicate that paracrine factors secreted by stem cells (including CSs) play an important role in cell‐mediated tissue regeneration 23, 24. These effects include but are not limited to: promotion of angiogenesis and cardiomyocyte proliferation and attenuation of apoptosis and fibrosis 25, 26, 27. Our hypothesis is that such mechanisms will not be seen in the ITB3‐blunted CS group as not sufficient cells were engrafted in the heart to drive such pathways. Our study also provided new evidence to support the notion that cell membranes and the adhesion molecules on then play essential roles in cell‐mediated tissue repair 28, 29.

## Conclusion

Our study demonstrated that integrin β‐3 plays an important role in cardiosphere attachment to fibronectin in the heart and governs the latter's engraftment and therapeutic benefit. Future strategies can be developed to overall express such integrins on cardiospheres to augment the therapeutic benefit of stem cell treatment.

## Conflict of interest

The authors declare no conflict of interest.
